# Molecular Epidemiology of Azole-Resistant *Aspergillus fumigatus* in France Shows Patient and Healthcare Links to Environmentally Occurring Genotypes

**DOI:** 10.3389/fcimb.2021.729476

**Published:** 2021-09-29

**Authors:** Steffi Rocchi, Thomas R. Sewell, Benoit Valot, Chloé Godeau, Audrey Laboissiere, Laurence Millon, Matthew C. Fisher

**Affiliations:** ^1^ Department of Parasitology and Mycology, Centre Hospitalier Universitaire, Besançon, France; ^2^ Chrono-Environnement Research Team UMR/CNRS-6249, Bourgogne-Franche-Comté University, Besançon, France; ^3^ Medical Research Council (MRC) Centre for Global Infectious Disease Analysis, Department of Infectious Disease Epidemiology, Imperial School of Public Health, Imperial College London, London, United Kingdom

**Keywords:** azole resistant, *Aspergillus fumigatus*, TR_34_/L98H, genetic relatedness, microsatellites

## Abstract

Resistance of the human pathogenic fungus *Aspergillus fumigatus* to antifungal agents is on the rise. However, links between patient infections, their potential acquisition from local environmental sources, and links to global diversity remain cryptic. Here, we used genotyping analyses using nine microsatellites in *A. fumigatus*, in order to study patterns of diversity in France. In this study, we genotyped 225 local *A. fumigatus* isolates, 112 azole susceptible and 113 azole resistant, collected from the Bourgogne-Franche-Comté region (Eastern France) and sampled from both clinical (*n* = 34) and environmental (*n* = 191) sources. Azole-resistant clinical isolates (*n* = 29) were recovered mainly from cystic fibrosis patients and environmental isolates (*n* = 84) from market gardens and sawmills. In common with previous studies, the TR_34_/L98H allele predominated and comprised 80% of resistant isolates. The genotypes obtained for these local TR_34_/L98H isolates were integrated into a broader analysis including all genotypes for which data are available worldwide. We found that dominant local TR_34_/L98H genotypes were isolated in different sample types at different dates (different patients and types of environments) with hospital air and patient’s isolates linked. Therefore, we are not able to rule out the possibility of some nosocomial transmission. We also found genotypes in these same environments to be highly diverse, emphasizing the highly mixed nature of *A. fumigatus* populations. Identical clonal genotypes were found to occur both in the French Eastern region and in the rest of the world (notably Australia), while others have not yet been observed and could be specific to our region. Our study demonstrates the need to integrate patient, healthcare, and environmental sampling with global databases in order to contextualize the local-scale epidemiology of antifungal resistant aspergillosis.

## Introduction

Fungal pathogens pose a growing threat to the health of humans, animals, ecosystems, food security, and the global economy, making their effective control necessary (Fisher et al., 2012). Azoles fungicides, with more than 30 compounds available (Fisher et al., 2018), are widely used in agriculture to protect cereal, vegetables, and vines from phytopathogenic fungi, as well as in the cultivation of ornamental plants and to preserve materials such as timber. These fungicides are used worldwide, particularly in Europe and Asia, where they represent one of the most commonly used classes of pesticides (Chowdhary et al., 2015; Chen et al., 2016). Due to their broad-spectrum action across the fungal kingdom, the azoles are also widely used as first-line drugs in medicine (human and animals) for the treatment of superficial and invasive fungal infections. Within this context of widespread use of antifungals across different compartments, the occurrence of multi-resistant pathogenic fungi represents an emerging threat to the control of plant, animal, and human diseases (Fisher et al., 2018). Recently reviewed (Verweij et al., 2020), substantial progress has been made towards building the argument that ecological “hotspots” occur where biotic and abiotic conditions converge to allow the growth of the thermophilic *Aspergillus fumigatus* in contact with sub-MIC concentrations of agricultural triazoles, generating conditions that are suitable for adaptation to drug pressure. Specifically, highly related genotypes of azole-resistant *A. fumigatus* have now been described in clinical and environmental samples, suggesting that humans are increasingly exposed to drug-resistant aerosolized *A. fumigatus* spores with broad public health consequences (Sewell et al., 2019; Rhodes et al., 2021).

Studying the population genetics and evolution of pathogens can help further our understanding of the evolution and spread of antimicrobial resistance, including tracing potential sources of inocula using molecular epidemiology. Specifically, for azole-resistant *A fumigatus* (AR*Af*), it is important to understand whether genotypes are randomly distributed or cluster geographically, and whether a common source of colonization may exist (van der Torre et al., 2021). In 2012, we described a first case of invasive aspergillosis caused by TR_34_/L98H AR*Af* in a farmer (Rocchi et al., 2014). In 2013, we described a second case, in a woodworker, who developed sinus aspergillosis due to TR_34_/L98H *A. fumigatus* (Jeanvoine et al., 2016). Motivated by these findings, the mycology team of Besançon (Eastern France) undertook a systematic screening of *A. fumigatus* to assess the burden of azole resistance across both clinical and environmental samples. In 2017, we observed an increase in the number of cystic fibrosis (CF) patients colonized with AR*Af*, with 16% of patients with *A. fumigatus*-positive cultures observed carrying the TR_34_/L98H resistance allele (Godeau et al., 2019) whereas previously, only one patient had this type of isolate in 2015. This percentage of resistance mirrors that reported by a British team (Abdolrasouli et al., 2018) and is among the highest occurrence in CF patients observed in European studies (Amorim et al., 2010; Mortensen et al., 2011; Burgel et al., 2012; Morio et al., 2012; Fischer et al., 2014; Prigitano et al., 2017; Guegan et al., 2018; Seufert et al., 2018; Lavergne et al., 2019).

Since 2017, a number of AR*Af* baring the TR_34_/L98H allele were detected in the air of Besançon hospital following fungal aerocontamination monitoring. Undertaken weekly using seven air sample locations in general hospital corridors, our surveillance has detected AR*Af* periodically between 2017 and 2019 (Rocchi et al., 2021). In parallel, two environmental studies were conducted in agricultural fields where azoles had been sprayed, in sawmills (Jeanvoine et al., 2017) and market gardens (Rocchi et al., 2018).

Across our team, we have collected 1,100 strains of the *Aspergilli* complex (*A. fumigatus* and cryptic species) isolated in our region (rural landscape in Eastern France). Here, we assess the genetic relatedness among a retrospective sample of 225 susceptible and resistant *A. fumigatus* from our collection for both clinical and environmental samples by comparing them to data (worldwide scale) held within pre-existing genotype databases.

## Materials and Methods

### Current Systematic Screening in Besançon Hospital

For both clinical and environmental samples, *A. fumigatus* were first isolated on malt agar media supplemented by itraconazole (2 mg L^−1^) or voriconazole (1 mg L^−1^) (van der Linden et al., 2013; Bader et al., 2015) at 37°C for clinical ones and 48°C for environmental ones. Subsequently, resistance was measured by concentration gradient Etest strip technique (Etest, bioMérieux, France) and/or checked by European Committee on Antimicrobial Susceptibility Testing (EUCAST) broth microdilution method (Arendrup et al., 2013). In case of azole-resistant isolate, a 495-bp section of the *beta-tubulin* gene (Glass and Donaldson, 1995) and the *cyp51A* gene and its promoter were amplified and sequenced (Mellado et al., 2001; Alanio et al., 2011).

PCRs were carried out in a 50-µl volume, containing 25 µl of GoTaq^®^ G2 Hot Start Green Master Mix, 2 µl of each set of primers (10 µM, **Table 1**), 50–100 ng of genomic DNA, and water (molecular biology quality) to 50 µl. Amplification was performed in a C1000 Touch™ thermal cycler (BIORAD) with 2 min at 95°C, and 34 cycles of 15 s at 95°C, 15 s at 61°C (for *beta-tubulin* primer sets), or 15 s at 55°C (for *cyp51A* primer sets), 10 s at 72°C, and one final cycle of 5 min at 72°C. The PCR products were analyzed by electrophoresis on 1% agarose gels, visualized with SYBR™ Safe DNA Gel Stain (ThermoFisher Scientific), and then purified (NucleoSpin^®^ Gel and PCR clean-up, Macherey-Nagel) to Sanger sequencing (Applied Biosystems 3130 Genetic Analyzer).

**Table 1:** Sequences of primers used for *beta-tubulin* and *cyp51A* and its promoter.

		Sequences	
*beta-tubulin*		Bt2a	5′-GGTAACCAAATCGGTGCTGCTTTC-3′
		Bt2b	5′-ACCCTCAGTGTAGTGACCCTTGGC-3′
*cyp51A* and its promoter	Set 1	PA5	5′-TCTCTGCACGCAAAGAAGAAC-3′
		PA7	5′-TCATATGTTGCTCAGCGG-3′
	Set 2	AF306F	5′-CACTGCAACTCTAATCCTCG-3′
		AF855R	5′-TAACGCAGACTGAGTCAAGC-3′
	Set 3	AF766F	5′-TTCGGATCGGACGTGGTGTA-3′
		AF1330R	5′-CGCTGATGGACGAAGACGAA-3′
	Set 4	AF1179F	5′-TGACGGTGACAAGGACTCTC-3′
		AF1709R	5′-ACAACCTCGTCGTTCTCCTG-3′
	Set 5	AF1426F	5′ AGTCTTCCTCCGCTCCAGTA-3′
		AF2025R	5′-ACACCTATTCCGATCACACC-3′

### Selection of Isolates for Genetic Relatedness Analysis

All AR*Af* obtained from April 2012 to December 2018, 113 isolates in total, were included in the genetic relatedness analysis. Azole-susceptible isolates (AS*Af*), isolated on azole or azole-free media, were retrospectively selected (see below) from the available collection according to the origin of resistant ones and to have groups with a comparable number of isolates between the different environments. For clinical isolates, especially those from CF patients, sensitivity to azoles was assayed using Etest strip technique and confirmed by EUCAST method for only one colony growing on medium with azole from sputum until 2018. For environmental analysis, sensitivity of all strains growing on medium with azole were assayed with EUCAST method and we inoculated the samples in parallel on azole free media (DG18). Due to the large number of *A. fumigatus* isolated on DG18, we selected only one isolate for every 10 growing on media to test their sensitivity. Isolates growing on medium with azoles but with EUCAST MIC > 2 µg/ml were stored as susceptible strains.

The majority of environmental resistant AR*Af* isolates came from market gardens (*n*=43), sawmills (*n*=24), and air of hospital (*n*=12). For sawmills and market gardens, we chose AS*Af* to match the location of AR*Af* isolates for each professional field (preferably within the same sample when possible, or in the same field or building for sawmills or, if not possible, on the same farm). In total, we analyzed a total of 63 isolates from market gardens (43 AR*Af* and 20 AS*Af*) and 65 from sawmills (24 AR*Af* and 42 AS*Af*).

With respect to isolates from hospital air sampling, as the detection of AR*Af* did not always coincide with an increase in the total number of *A. fumigatus* in hospital corridors, we selected 45 AS*Af* in the following way: AS*Af* isolated from a contamination peak in June 2017, localized in one hospitalization ward (after a heating system malfunction), with a selection of 24 AS*Af* in six rooms and bathrooms of the department. AS*Af* were isolated during a general contamination peak in December 2018 (68 colony forming units/m^3^ of air) in the general corridors of the hospital where we selected 12 AS*Af*. Six ASA*f* were isolated in June 2018, on the same day as a TR_34_/L98H AR*Af* and three AS*Af* were isolated the following week.

**Figure 1:**
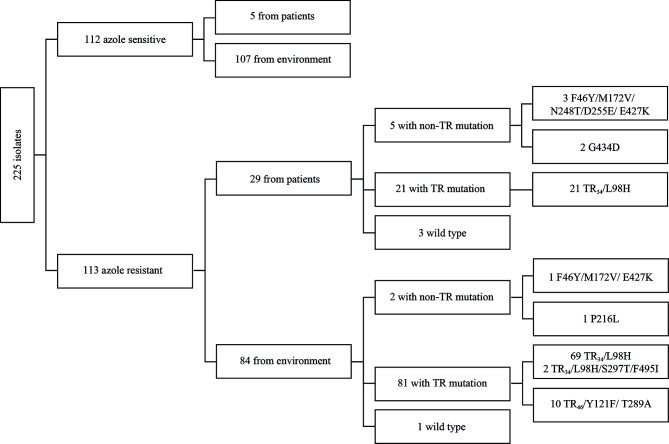
Diagram with type of sampling, azole resistance, and *cyp51A* mutations of analyzed isolates.

In total, we analyzed 112 AS*Af* coming from 5 clinical and 107 environmental samples and 113 AR*Af* coming from 29 clinical and 84 environmental samples (**Figure 1**).

### Genotyping Method

Microsatellite genotyping by short tandem repeats for *A. fumigatus* (STR*Af*) assay (de Valk et al., 2005) was performed to assess the genetic relatedness between 225 susceptible and resistant *A. fumigatus*.

We modified the method developed by de Valk et al. (2005) by changing the fluorochrome TET (tetrachlorofluorescein) to NED (2.7’,8’-benzo-5’-fluoro-2’,4,7-tricloro-5-carboxyfluorescein). Diluted PCR products were combined with GeneScan™ 400 HD ROX™ size standard (ThermoFisher, Waltham, MA, USA) for fragment detection by capillary electrophoresis (Applied Biosystems 3130 Genetic Analyzer device, ThermoFisher, Waltham, MA, USA).

Peaks were visualized with Microsatellite analysis application on ThermoFisher cloud dashboard (https://apps.thermofisher.com/apps/spa/#/dashboard). For each marker, the correct peak was selected and transformed into a repeat number based on the Af293 STR*Af* genotype. The number of repeats was inferred from the available genome for this strain by an automated script using R Studio software.

### Data Analysis

Microsatellite genotype distances were calculated using Bruvo’s distance (Bruvo et al., 2004). The analyses were done in three steps. Firstly, all susceptible and resistant isolates from the mycology team of Besançon were used. Genotypic richness was assessed by the total number of unique multilocus genotypes (MLGs) or expected MLGs (eMLGs), which approximates the number of genotypes that would be expected at the largest, shared sample size based on rarefaction. Diversity (Simpson’s, Shannon–Wiener, or Stoddart and Taylor’s Indexes) and evenness were also calculated for each type of isolate (azole susceptible and resistant) with the R package poppr (Kamvar et al., 2015).

Secondly, only local TR_34_/L98H isolates were analyzed in order to see the proximity according to their origin. Thirdly, we compared our local TR_34_/L98H isolates to those with available data worldwide. For this, we used a global dataset housed within the online AfumID application (https://afumid.shinyapps.io/afumID/) (Sewell et al., 2019). We thus included a further 194 isolates that had previously been isolated and genotyped from 16 countries. In order to ensure harmonization, the number of alleles was recalibrated between the two databases using the reference strain Af293.

Data were represented by circular genetic tree build by a Neighbor-Joining method clustering from a matrix of bruvo distance (Bruvo et al., 2004) using ape library (Paradis and Schliep, 2019) and interactive tree of life (iTOL) software (https://itol.embl.de/) and by minimum spanning networks, calculated *via* the bruvo.msn function on the poppr library (Kamvar et al., 2015).

## Results

### *Cyp51A* Gene Mutations

**Figure 2:**
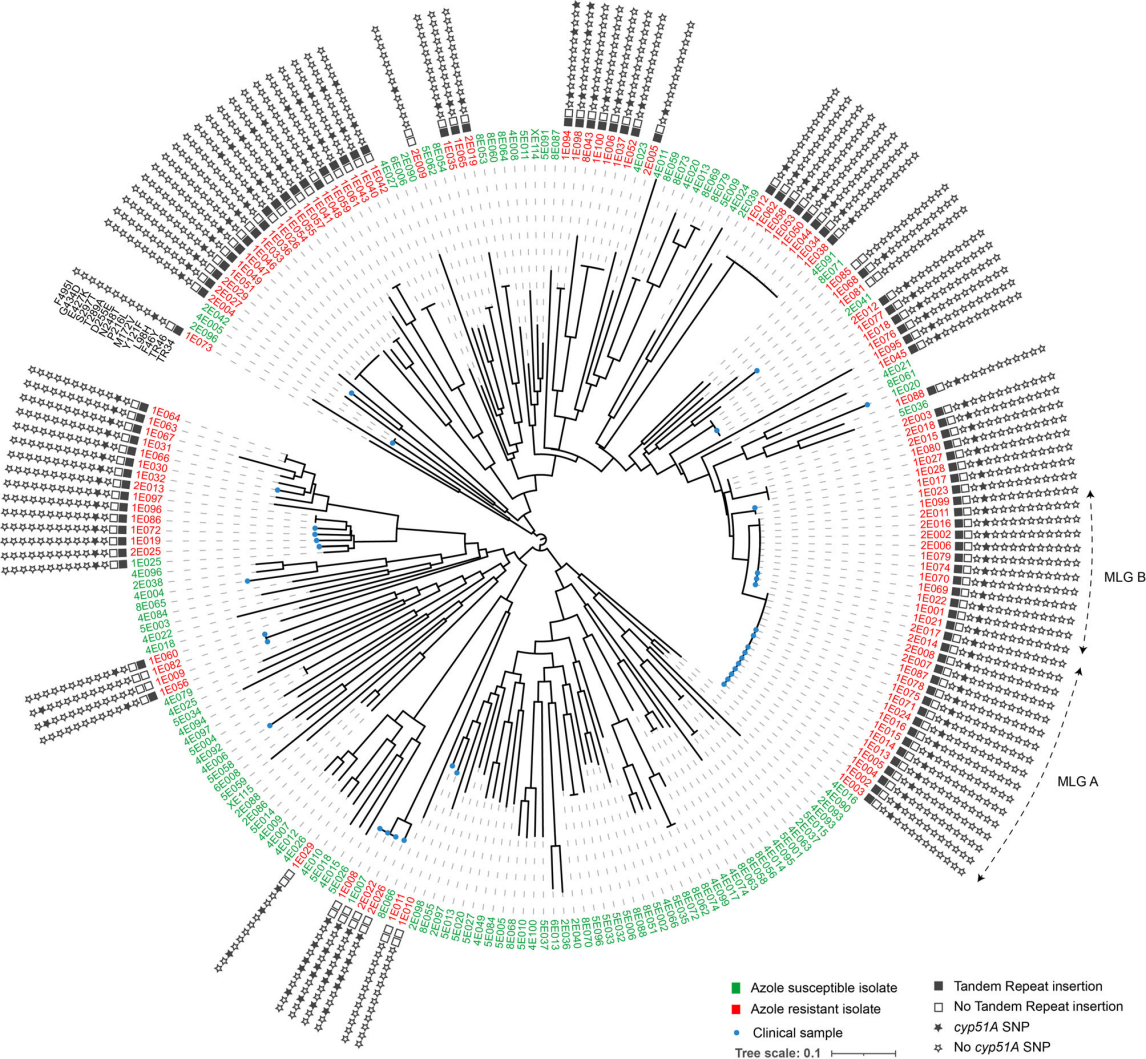
Circular dendrogram illustrating relationship among 113 azole-resistant (with details of tandem repeat and *cyp51A* SNP) and 112 azole-susceptible *Aspergillus fumigatus*, isolated from clinical or environmental samples. Genetic relationships are based on short tandem repeat at nine loci, using Bruvo’s distance and Neighbor-Joining clustering. The two most common multilocus genotypes (MLG) are denoted by the letters A and B Numbers are ID isolates. The stars provide information on the TR or SNP present on the *cyp51A* gene.

Among the 113 AR*Af* that were isolated, 90 isolates contained the TR_34_/L98 *cyp51A* allele, 2 TR_34_/L98/S297T/F495I, 10 TR_46_/Y121F/T289A, and 7 non-TR allele (F46Y/M172V/N248T/D255E/E427K, F46Y/M172V/E427K, P216L, or G434D). Four AR*Af* (1E010, 1E011, and 1E081 from clinical samples and 1E085 from an environmental sample, **Figure 2** and **Supplementary Table data**) did not contain any polymorphisms in the *cyp51A* gene.

### Genotyping Results: Besançon Local Scale

The total number of MLGs was higher in the sensitive group than in the resistant group with 102 MLGs found in the 112 AS*Af* isolates and 53 MLGs observed among the 113 barcoded AR*Af*.

When comparing the two most abundant groups (AS*Af* and TR_34_/L98H AR*Af*), it was apparent that genotypic richness, represented by the total number of MLG or expected MLG (eMLG), was lower in the TR_34_/L98H AR*Af* group (**Table 2**). Regardless of the index used (Simpson’s, Shannon–Wiener, or Stoddart and Taylor’s Indexes), the genotype diversity was also lower in the TR_34_/L98H AR*Af* group. The evenness measure (**Table 2**) indicates that the MLGs observed in the AS*Af* population are closer to equal abundance (which would be equal to 1) than those in the TR_34_/L98H AR*Af* population. Indeed, when we look at the distribution of MLGs for each group, there are many more unique AS*Af* profiles than AR*Af* profiles.

**Table 2:** Genotypic richness, diversity, and evenness information for azole-susceptible and azole-resistant TR_34_/L98H isolates.

Population	*N*	MLG	eMLG	Λ	*H*	*G*	E.5
Azole-susceptible isolates	112	102	9.93	0.989	4.59	95.03	0.960
Azole-resistant TR_34_/L98H isolates	90	37	7.80	0.923	3.07	12.98	0.583

N, Number of individuals observed; MLG, Number of multilocus genotypes observed; eMLG, The number of expected MLG at the smallest sample size based on rarefaction; λ, Simpson’s Index; H, Shannon–Wiener Index; G, Stoddart and Taylor’s Index; E.5, Evenness mesure.

When one MLG was shared by isolates of AS*Af*, it was only shared by two isolates and from a similar source (clinical or environmental). Only one MLG was found both in a clinical isolate (1E005, CF patient June 2017) and in an environmental isolate (2E042, hospital air June 2018).

One MLG was shared by sensitive and resistant *A. fumigatus* isolated from one CF patient: one susceptible (1E007) and two resistant (2E022 and 1E008) isolates. These three strains had F46Y/M172V/N248T/D255E/E427K polymorphism in *cyp51A*.

**Figure 3:**
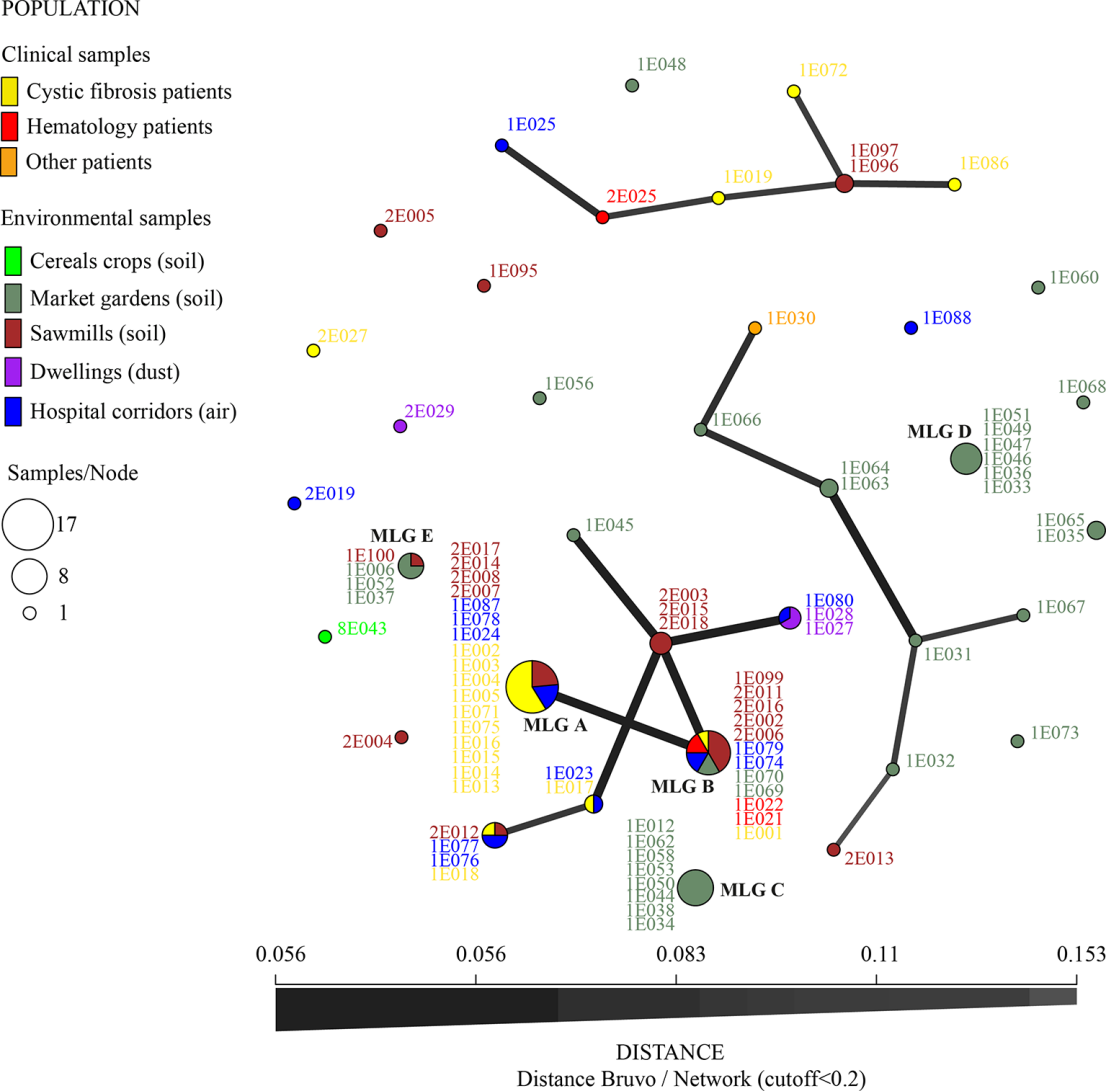
Minimum spanning network for TR34/L98H isolates (nine microsatellite loci STRAf using Bruvo’s distance, with distance cutoff < 0.2). Colors represent the origin of sampling and numbers are ID isolates. Link thickness is proportional to genotype similarity. The most common multilocus genotypes (MLG) are denoted by the letters A, B, C, D, and E.

For AR*Af*, five MLGs corresponding to TR_34_/L98H allele were share by several isolates (**Table 3**). Two main MLGs (A and B in **Figure 2**), differing only by a single repeat at the STR*Af* 3A loci and corresponding to 17 and 12 isolates, respectively, were shared by isolates recovered from both clinical and environmental samples. In addition to being isolated both in the clinic and in the environment, these isolates were also widely found in different environments across our region and also from different categories of patients or date of sampling (**Figure 3** and **Table 3**).

**Table 3:** Multilocus genotypes (MLG) shared by isolates with TR_34_/L98H allele.

MLG	Origin of sample	Type of patient or environment	Date of isolation	ID
MLG A (17 isolates)	Clinic	Cystic fibrosis patient	09/09/2015 ^a^	1E002
			13/12/2016 ^a^	1E003
			10/01/2017 ^b^	1E014
				1E015
			12/01/2017	1E016
			17/01/2017	1E004
			11/07/2017	1E013
			12/07/2017	1E005
			14/09/2017 ^a^	1E071
			10/11/2017 ^a^	1E075
	Environment	Hospital (air)	11/06/2015	1E024
			17/11/2017	1E078
			10/04/2018	1E087
		Sawmill (soil)	24/11/2014	2E007
			16/02/2015	2E017
			09/02/2016	2E008
			06/04/2016	2E014
MLG B (12 isolates)	Clinic	Hematology	27/04/2012	1E021
			11/05/2012	1E022
		Cystic fibrosis	04/10/2016 ^a^	1E001
	Environment	Hospital (air)	17/10/2017	1E074
			21/11/2017	1E079
		Sawmill (soil)	24/11/2014	2E006
			14/01/2016 ^c^	1E099
				2E002
			31/03/2016	2E016
			21/04/2016	2E011
		Market garden (soil)	10/03/2017 ^c^	1E069
				1E070
MLG C (8 isolates)	Environment	Market garden (soil)	07/03/2017 ^d^	1E012
				1E034
				1E038
				1E044
				1E050
				1E053
				1E058
				1E062
MLG D (6 isolates)	Environment	Market garden (soil)	07/03/2017 ^d^	1E033
				1E036
				1E046
				1E047
				1E049
				1E051
MLG E (4 isolates)	Environment	Sawmill (soil)	14/01/2016	1E100
		Market garden (soil)	07/03/2017 ^c^	1E006
				1E037
				1E052

Date of isolation: Day/Month/Year.

a Same cystic fibrosis chronically colonized APBA patient.

b Two different cystic fibrosis patients.

c In the same sawmill or market garden but different soil samples.

d Same market garden, in six different soil samples coming from two different sites found 1.5 km apart.

Two highly dissimilar MLGs of eight and six isolates (MLG C and D, **Figure 3**) were also found in the same market garden, in six different soil samples coming from two different sites where difenoconazole was used to treat vegetable fields. These fields were found 1.5 km apart. In this market garden, we also recovered 14 different MLGs, with one MLG (MLG E, **Figure 3**) shared with one sawmill 40 km away (all green MLG in **Figure 3**, except MLG B and 1E073 MLG, which were found in another market garden).

### Genotyping Results for TR_34_/L98H Isolates: Global Scale

**Figure 4:**
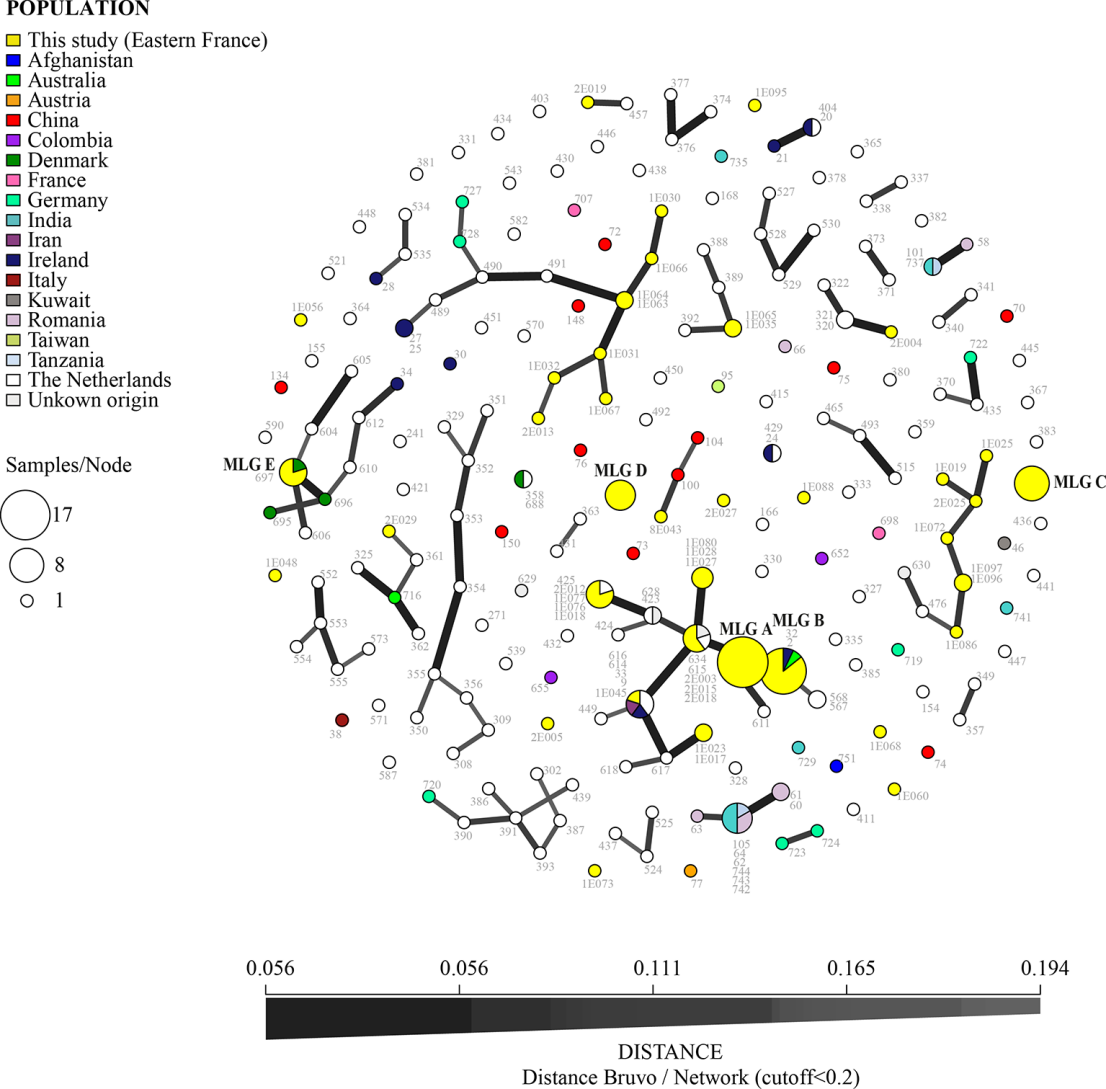
Minimum spanning network for TR_34_/L98H isolates (nine microsatellite loci STR*Af* using Bruvo’s distance, with distance cutoff < 0.2) from Besançon mycology team and from the world (AfumID application). Colors represent the origin of sampling and numbers are ID isolates. Link thickness is proportional to genotype similarity. The most common multilocus genotypes (MLGs) found in Besançon laboratory are denoted by the letters A, B, C, D, and E.

A subset of the dominant MLGs in the Franche-Comté area were commonly shared with isolates found worldwide: MLG B (found in CF patients, hospital air, sawmills, and market gardens) was also detected in Australia and Ireland and MLG E (detected in market gardens and sawmills) was also isolated in Denmark (**Figure 4**). In comparison, MLG A, C, and D, while common in our dataset, were not observed in the global dataset, suggesting that they are geographically spatially constrained.

Other MLGs found in Eastern France were also closely related to those occurring more widely including those found in the Netherlands (links between the MLG on the **Figure 4**). One MLG was shared by four isolates in Besançon (a sawmill, twice in hospital air and once in a CF patient) and one isolate in the Netherlands. Another MLG is common with Ireland and Iran isolates and one is common with the Netherlands and another country (not known in the AfumID application). Nine MLG corresponding to nine isolates (five from market gardens, two from sawmills, one from hospital air, and one from CF patient) appear to be completely different (no link in **Figure 4** with fixed cutoff) and were only found in the Besançon collection of isolates

## Discussion

In our study, we describe linked patient sampling, healthcare bioaerosol monitoring, and environmental sampling of *A. fumigatus* in order to explore potential links using molecular epidemiology. STR*Af* genotyping that we undertook demonstrated spatial heterogeneity, with some clones occurring far more widely than others. These data highlight the importance of incorporating spatial scale towards understanding of the local population genetic structure of *A. fumigatus* and the epidemiology AR*Af*.

Currently, the modes of dissemination, the occurrence of resistant genotypes in the environment, and the likelihood of exposure to at-risk patients remain poorly known. When applied locally, genotyping methods can shed light into the source of infection with various studies showing possible nosocomial acquisition of *A. fumigatus* and potentially AR*Af* (van der Torre et al., 2021). Three studies have suggested that dissemination from patient to patient occurs, as well as from patient to the healthcare environment (Pegues et al., 2002; Lemaire et al., 2018; Engel et al., 2019). For animals, the dynamics of resistant animal populations in livestock in relation to the environment (except for poultry) are poorly described and are unknown in wildlife. In both cases, it is likely that exposure to AR*Af* genotypes is higher for individuals in environment that are frequently exposed to fungicides and/or also to organic matter (leaf litter, compost, and cultivated soils rich in organic matter). Clearly, studies to assess the extent that resistant genotypes are stratified by region and environment will lend nuanced understanding to the ecological processes that are thought to generate AR*Af.*

In this study, as in others, we observe a reduced genetic diversity of TR_34_/L98H isolates (the most common resistance mechanism) compared to susceptible strains, suggesting a more recent emergence of this genotype (Camps et al., 2012). This allele is suggested to have emerged around 1997, perhaps in the Netherlands and dispersed across Europe, although other regions of origin are possible (Snelders et al., 2012; Abdolrasouli et al., 2015; Bader et al., 2015; Garcia-Rubio et al., 2018). Currently, TR_34_/L98H is increasingly described in other global settings and the occurrence of TR_34_/L98H genotypes does not seem to be limited to Europe. Aerial dispersal is most likely a major contributor to the long-distance migration of airborne *A. fumigatus* conidia owing to the widespread detection of aerosolized *A. fumigatus* spores (Shelton et al., 2020), which can also be moved in traded products (Dunne et al., 2017). Indeed, identical genotypes were described in several centers and countries, sometimes separated by thousands of kilometres (Chowdhary et al., 2012; Sewell et al., 2019). In 2012, a study of environmental isolates from Iran reported a TR_34_/L98H MLG closely related to Dutch and Indian isolates (Badali et al., 2013). A recent Japanese publication described TR_34_/L98H isolates clustering with strains from the Netherlands and France (Tsuchido et al., 2019).

In our study, one of the two dominant MLGs (MLG B) isolated in different environments in the Besançon region and isolated from different patients over several years was identical to an MLG in Australia and Ireland. Owing to the highly diverse nature of the STR*Af* barcoding loci, the likelihood by these MLGs being identical by chance rather than by descent is vanishingly unlikely (Sewell et al., 2019). Conversely, we have also found MLGs that have so far not been found in other countries. This is particularly the case for MLG A, which has high frequency in varied environments (sawmills and hospital air) as well as in different patients over several years. In Taiwan, a team has observed a potential global spread of TR_34_/L98H isolates but also a local genetic clustering of some clinical and environmental TR_34_/L98H (Wang et al., 2018). Other works have also reported local clusters, as in China with clinical and environmental TR_34_/L98H/S297T/F495I isolates (Deng et al., 2017; Chen et al., 2018). The two isolates carrying this mutation found in the French sawmill do not have the same STR*Af* profiles as those described in these works. In Germany, local factors have been evoked as the reason for the increased prevalence of specific genotypes (Bader et al., 2015). Resistance could therefore also result from the appearance of different clones located in different countries or regions when there is sufficient selection pressure. This could explain the greater diversity of genotypes found in vegetable crops in the market gardens, for example.

In our study, we have shown that AR*Af* occurs in the air of hospital and, in this case, could be acquired by patients during episodes of immunosuppression. In our hospital, resistant isolates were found in the general corridors, not in the hematology departments, thus reducing the risk of acquiring invasive aspergillosis caused by a resistant genotype. However, we described MLGs found in CF patients and in environments such as hospital air, and also with hospital visits coinciding with the isolation of certain resistant genotypes; this is the case, for example, for MLGs A and B. However, the wide distribution of these MLGs, both locally for MLG A (different environments) and globally for MLG B (different environments and in other countries), prevents us from conclusively asserting nosocomial acquisition of these isolates in CF patients. This is because, on a probabilistic basis, there is a non-negligible likelihood that these dominant genotypes may have been acquired through exposures outside of the hospital. However, a single MLG found only in Besançon was isolated from hospital air (1E023) and from sputum of a CF patient (1E017) 1.5 months apart. Even if we do not have the analytical power to definitively prove this, the potential for nosocomial contamination of the healthcare environment cannot currently be excluded.

Microsatellite genotyping is widely used in eukaryotes. In *A. fumigatus*, it has been used to identify the potential origins of resistant genotypes, and how genotypes are shared between patients, geographical regions, countries, and continents (Korfanty et al., 2019). Genotyping analyses with the nine-microsatellite-based method in *A. fumigatus* (de Valk et al., 2005) provide high discriminatory power and reproducibility (Klaassen and Osherov, 2007). For our local isolate comparisons, we can be sure that MLG A and B of the Besançon region isolates are different, as they were analyzed with the same methodology (PCR, fragment analysis, and copy number transformation). However, when we compare the profiles of isolates analyzed in different laboratories, how can we be sure that two isolates with only one microsatellite difference, for example, are indeed different or that the difference is due to interlaboratory variations? MLG A (only local isolates) and MLG B (local and found in Australia and Ireland) only differ by one repeat on a microsatellite, STR*Af* 3A, which is the loci showing the greatest diversity, as has already been shown for agricultural strains (Ashu et al., 2018). It is therefore important to secure or find ways to harmonize methods of analysis on a large scale so that results can be reliably compared, and ensure in our case that it is indeed the MLG A that is local and that the MLG B has a global distribution. Interlaboratory controls should also be considered with, in particular, the implementation of standardization of sizing data by using allelic ladders (de Valk et al., 2009). Moreover, microsatellite genotyping does not provide sufficiently fine-grained information on the degree of proximity of two strains with two MLG, for example, whereas whole genome sequencing (WGS) can (Hagiwara et al., 2014; Rhodes et al., 2021). High-throughput WGS techniques are now poised to surpass other typing methods because of the high resolution of the data generated and the decreasing costs as performance increases. Better genetic knowledge of resistant strains through genomic approaches will help define the population structure of *A. fumigatus* and inform the future evolutionary trajectory of resistant phenotypes.

While WGS techniques have a higher analysis resolution than other genotyping techniques, the STR*Af* method, coupled with mutation screening and even mating type characterization, appears to be a good compromise to rapidly and simply screen relatively large numbers of isolates that have resulted from surveillance programs (van der Torre et al., 2021). Then, as in our case, where a “super clone” emerges from the analysis, it would be judicious to use WGS analysis to refine analyses in order to further explore patient–environmental links. Going forward, there is a need to continue to study the environmental conditions (both in host and in the environment) that facilitate the development, selection, and spread of resistance genotypes with large-scale multidisciplinary collaborations.

## Data Availability Statement

The raw data supporting the conclusions of this article will be made available by the authors, without undue reservation.

## Author Contributions

Conceptualization: SR and MF. Molecular analysis: SR, CG, and AL. Data analysis, SR, TS, and BV. Writing—original draft preparation: SR. Writing, review, and editing: SR, MF, TS, CG, BV, and LM. Funding acquisition: SR and LM. All authors contributed to the article and approved the submitted version.

## Funding

The routine analyses were carried out within the framework of hospital activity, without external funding. The in-depth analysis of resistant strains was financially supported by LTSER “Zone Atelier Arc Jurassien”, “Observatoire des Sciences de l’Univers Terre Homme Environnement Temps Astronomie”, Bourgogne Franche-Comté-University, and RECOTOX network supported by AllEnvi and co-carried by INRAE and CNRS-INEE. MF and TS were supported by the UK Medical Research Council and the UK Natural Environmental Research Council.

## Conflict of Interest

The authors declare that the research was conducted in the absence of any commercial or financial relationships that could be construed as a potential conflict of interest.

## Publisher’s Note

All claims expressed in this article are solely those of the authors and do not necessarily represent those of their affiliated organizations, or those of the publisher, the editors and the reviewers. Any product that may be evaluated in this article, or claim that may be made by its manufacturer, is not guaranteed or endorsed by the publisher.
