# Supplementary material for: Molecular Epidemiology of Azole-Resistant Aspergillus fumigatus in France Shows Patient and Healthcare Links to Environmentally Occurring Genotypes
Source: Front Cell Infect Microbiol. 2021 Sep 29;11:729476. doi: 10.3389/fcimb.2021.729476 (PMC8512841; doi:10.3389/fcimb.2021.729476)
Supplement: Supplementary file 1 [file Table_1.docx]

Table S1 : Details of local isolates chronologically arranged (Mycology team Besançon, France).

| **ID** | **Date of isolation** | **Origine of sample** | **Type of patient or environment** | **Sensitive (S) or Resistant** | **Mutation cyp51A gene** | **Short tandem repeats for**  ***A. fumigatus* (STR*Af*)** | | | | | | | | |
| --- | --- | --- | --- | --- | --- | --- | --- | --- | --- | --- | --- | --- | --- | --- |
|  |  |  |  |  |  | **2A** | **2B** | **2C** | **3A** | **3B** | **3C** | **4A** | **4B** | **4C** |
| 1E021 | 27/04/2012 | clinic | hematology | R | TR34/L98H | 14 | 21 | 8 | 31 | 8 | 6 | 8 | 10 | 20 |
| 1E022 | 11/05/2012 | clinic | hematology | R | TR34/L98H | 14 | 21 | 8 | 31 | 8 | 6 | 8 | 10 | 20 |
| 8E043 | 06/05/2013 | environment | cereals crops (soil) | R | TR34/L98H | 23 | 10 | 9 | 10 | 8 | 10 | 8 | 10 | 8 |
| 1E020 | 02/08/2013 | clinic | other patient | S | NR | 27 | 21 | 12 | 30 | 8 | 6 | 8 | 10 | 19 |
| 1E030 | 10/10/2013 | clinic | other patient | R | TR34/L98H | 23 | 21 | 16 | 99 | 11 | 7 | 17 | 9 | 10 |
| 2E006 | 24/11/2014 | environment | sawmill (soil) | R | TR34/L98H | 14 | 21 | 8 | 31 | 8 | 6 | 8 | 10 | 20 |
| 2E007 | 24/11/2014 | environment | sawmill (soil) | R | TR34/L98H | 14 | 21 | 8 | 30 | 8 | 6 | 8 | 10 | 20 |
| 4E090 | 24/11/2014 | environment | sawmill (soil) | S | NR | 19 | 19 | 8 | 15 | 8 | 22 | 8 | 9 | 5 |
| 4E091 | 24/11/2014 | environment | sawmill (soil) | S | NR | 14 | 20 | 8 | 32 | 8 | 10 | 8 | 10 | 36 |
| 5E010 | 21/01/2015 | environment | sawmill (soil) | S | NR | 19 | 12 | 16 | 25 | 9 | 18 | 9 | 8 | 7 |
| 2E017 | 16/02/2015 | environment | sawmill (soil) | R | TR34/L98H | 14 | 21 | 8 | 30 | 8 | 6 | 8 | 10 | 20 |
| 4E092 | 16/02/2015 | environment | sawmill (soil) | S | NR | 18 | 19 | 10 | 28 | 9 | 35 | 20 | 11 | 5 |
| 4E093 | 16/02/2015 | environment | sawmill (soil) | S | NR | 18 | 19 | 8 | 26 | 22 | 21 | 16 | 9 | 7 |
| 4E094 | 16/02/2015 | environment | sawmill (soil) | S | NR | 18 | 25 | 17 | 31 | 10 | 7 | 18 | 10 | 8 |
| 4E095 | 16/02/2015 | environment | sawmill (soil) | S | NR | 18 | 19 | 8 | 35 | 13 | 20 | 9 | 9 | 5 |
| 1E024 | 11/06/2015 | environment | hospital (air) | R | TR34/L98H | 14 | 21 | 8 | 30 | 8 | 6 | 8 | 10 | 20 |
| 1E002 | 09/09/2015 | clinic | cystic fibrosis | R | TR34/L98H | 14 | 21 | 8 | 30 | 8 | 6 | 8 | 10 | 20 |
| 1E096 | 14/01/2016 | environment | sawmill (soil) | R | TR34/L98H | 23 | 21 | 16 | 119 | 8 | 7 | 5 | 9 | 10 |
| 1E097 | 14/01/2016 | environment | sawmill (soil) | R | TR34/L98H | 23 | 21 | 16 | 119 | 8 | 7 | 5 | 9 | 10 |
| 1E098 | 14/01/2016 | environment | sawmill (soil) | R | TR34/L98H/S297T/F495I | 14 | 10 | 9 | 40 | 9 | 11 | 8 | 10 | 20 |
| 1E099 | 14/01/2016 | environment | sawmill (soil) | R | TR34/L98H | 14 | 21 | 8 | 31 | 8 | 6 | 8 | 10 | 20 |
| 1E100 | 14/01/2016 | environment | sawmill (soil) | R | TR34/L98H | 23 | 10 | 9 | 10 | 9 | 6 | 8 | 10 | 20 |
| 2E002 | 14/01/2016 | environment | sawmill (soil) | R | TR34/L98H | 14 | 21 | 8 | 31 | 8 | 6 | 8 | 10 | 20 |
| 2E003 | 14/01/2016 | environment | sawmill (soil) | R | TR34/L98H | 14 | 21 | 8 | 32 | 8 | 6 | 8 | 10 | 20 |
| 4E096 | 14/01/2016 | environment | sawmill (soil) | S | NR | 23 | 23 | 15 | 39 | 10 | 45 | 9 | 9 | 8 |
| 4E097 | 14/01/2016 | environment | sawmill (soil) | S | NR | 18 | 25 | 17 | 31 | 10 | 7 | 18 | 10 | 8 |
| 5E032 | 14/01/2016 | environment | sawmill (soil) | S | NR | 18 | 12 | 8 | 25 | 10 | 21 | 8 | 9 | 7 |
| 5E033 | 14/01/2016 | environment | sawmill (soil) | S | NR | 18 | 12 | 16 | 14 | 9 | 22 | 8 | 9 | 7 |
| 5E034 | 14/01/2016 | environment | sawmill (soil) | S | NR | 18 | 20 | 15 | 29 | 11 | 20 | 26 | 26 | 8 |
| 5E035 | 14/01/2016 | environment | sawmill (soil) | S | NR | 19 | 12 | 11 | 25 | 8 | 20 | 17 | 8 | 5 |
| 5E036 | 14/01/2016 | environment | sawmill (soil) | S | NR | 25 | 21 | 8 | 43 | 8 | 6 | 8 | 10 | 19 |
| 5E037 | 14/01/2016 | environment | sawmill (soil) | S | NR | 18 | 12 | 14 | 27 | 10 | 14 | 9 | 11 | 7 |
| 2E005 | 27/01/2016 | environment | sawmill (soil) | R | TR34/L98H | 13 | 20 | 9 | 23 | 9 | 8 | 8 | 10 | 19 |
| 2E015 | 27/01/2016 | environment | sawmill (soil) | R | TR34/L98H | 14 | 21 | 8 | 32 | 8 | 6 | 8 | 10 | 20 |
| 5E062 | 27/01/2016 | environment | sawmill (soil) | S | NR | 25 | 20 | 12 | 35 | 8 | 7 | 8 | 10 | 22 |
| 5E020 | 04/02/2016 | environment | sawmill (soil) | S | NR | 18 | 12 | 17 | 27 | 9 | 20 | 8 | 8 | 5 |
| 5E026 | 04/02/2016 | environment | sawmill (soil) | S | NR | 10 | 17 | 12 | 20 | 12 | 13 | 7 | 5 | 6 |
| 5E058 | 04/02/2016 | environment | sawmill (soil) | S | NR | 21 | 23 | 18 | 26 | 9 | 14 | 9 | 13 | 8 |
| 2E008 | 09/02/2016 | environment | sawmill (soil) | R | TR34/L98H | 14 | 21 | 8 | 30 | 8 | 6 | 8 | 10 | 20 |
| 5E011 | 09/02/2016 | environment | sawmill (soil) | S | NR | 13 | 20 | 11 | 33 | 8 | 11 | 8 | 9 | 19 |
| 6E013 | 09/02/2016 | environment | sawmill (soil) | S | NR | 18 | 12 | 12 | 25 | 20 | 19 | 14 | 8 | 7 |
| 2E018 | 17/02/2016 | environment | sawmill (soil) | R | TR34/L98H | 14 | 21 | 8 | 32 | 8 | 6 | 8 | 10 | 20 |
| 4E099 | 17/02/2016 | environment | sawmill (soil) | S | NR | 19 | 12 | 11 | 15 | 8 | 18 | 17 | 9 | 5 |
| 5E013 | 17/02/2016 | environment | sawmill (soil) | S | NR | 18 | 12 | 11 | 16 | 9 | 13 | 8 | 9 | 5 |
| 5E014 | 17/02/2016 | environment | sawmill (soil) | S | NR | 25 | 22 | 20 | 29 | 8 | 28 | 10 | 9 | 5 |
| 5E084 | 25/02/2016 | environment | sawmill (soil) | S | NR | 17 | 12 | 16 | 14 | 10 | 17 | 8 | 9 | 5 |
| 1E094 | 01/03/2016 | environment | sawmill (soil) | R | TR34/L98H/S297T/F495I | 14 | 10 | 9 | 40 | 9 | 11 | 8 | 10 | 20 |
| 1E095 | 01/03/2016 | environment | sawmill (soil) | R | TR34/L98H | 14 | 24 | 8 | 32 | 8 | 32 | 10 | 9 | 20 |
| 5E018 | 01/03/2016 | environment | sawmill (soil) | S | NR | 10 | 14 | 10 | 16 | 12 | 16 | 7 | 5 | 6 |
| 2E012 | 10/03/2016 | environment | sawmill (soil) | R | TR34/L98H | 14 | 21 | 8 | 33 | 8 | 10 | 8 | 10 | 20 |
| 4E049 | 10/03/2016 | environment | sawmill (soil) | S | NR | 18 | 12 | 11 | 26 | 10 | 21 | 8 | 8 | 5 |
| 4E084 | 17/03/2016 | environment | sawmill (soil) | S | NR | 23 | 19 | 15 | 49 | 10 | 7 | 13 | 9 | 5 |
| 2E004 | 23/03/2016 | environment | sawmill (soil) | R | TR34/L98H | 25 | 10 | 8 | 85 | 8 | 12 | 8 | 7 | 5 |
| 5E059 | 23/03/2016 | environment | sawmill (soil) | S | NR | 25 | 19 | 20 | 26 | 18 | 17 | 10 | 16 | 8 |
| 5E015 | 25/03/2016 | environment | sawmill (soil) | S | NR | 21 | 19 | 8 | 26 | 9 | 21 | 15 | 9 | 5 |
| 2E016 | 31/03/2016 | environment | sawmill (soil) | R | TR34/L98H | 14 | 21 | 8 | 31 | 8 | 6 | 8 | 10 | 20 |
| 4E074 | 31/03/2016 | environment | sawmill (soil) | S | NR | 20 | 12 | 9 | 10 | 7 | 10 | 8 | 9 | 10 |
| 4E079 | 31/03/2016 | environment | sawmill (soil) | S | NR | 18 | 23 | 16 | 26 | 10 | 27 | 22 | 10 | 7 |
| 4E100 | 31/03/2016 | environment | sawmill (soil) | S | NR | 19 | 12 | 15 | 27 | 9 | 17 | 9 | 8 | 7 |
| 5E027 | 31/03/2016 | environment | sawmill (soil) | S | NR | 18 | 12 | 16 | 26 | 9 | 20 | 8 | 8 | 5 |
| 2E013 | 06/04/2016 | environment | sawmill (soil) | R | TR34/L98H | 23 | 21 | 16 | 108 | 11 | 7 | 18 | 9 | 10 |
| 2E014 | 06/04/2016 | environment | sawmill (soil) | R | TR34/L98H | 14 | 21 | 8 | 30 | 8 | 6 | 8 | 10 | 20 |
| 4E063 | 06/04/2016 | environment | sawmill (soil) | S | NR | 18 | 19 | 8 | 26 | 9 | 20 | 9 | 9 | 5 |
| 4E066 | 06/04/2016 | environment | sawmill (soil) | S | NR | 19 | 12 | 11 | 25 | 8 | 21 | 17 | 8 | 5 |
| 5E001 | 06/04/2016 | environment | sawmill (soil) | S | NR | 18 | 19 | 8 | 35 | 13 | 20 | 9 | 9 | 5 |
| 5E002 | 06/04/2016 | environment | sawmill (soil) | S | NR | 18 | 12 | 8 | 29 | 8 | 19 | 9 | 9 | 5 |
| 4E004 | 20/04/2016 | clinic | cystic fibrosis | S | NR | 23 | 23 | 14 | 39 | 10 | 47 | 10 | 9 | 8 |
| 2E009 | 21/04/2016 | environment | sawmill (soil) | R | P216L | 26 | 20 | 11 | 33 | 8 | 7 | 8 | 10 | 30 |
| 2E011 | 21/04/2016 | environment | sawmill (soil) | R | TR34/L98H | 14 | 21 | 8 | 31 | 8 | 6 | 8 | 10 | 20 |
| 5E091 | 21/04/2016 | environment | sawmill (soil) | S | NR | 13 | 20 | 11 | 34 | 8 | 10 | 8 | 10 | 20 |
| 5E096 | 21/04/2016 | environment | sawmill (soil) | S | NR | 18 | 12 | 17 | 25 | 9 | 24 | 8 | 9 | 7 |
| 6E006 | 21/04/2016 | environment | sawmill (soil) | S | NR | 25 | 20 | 11 | 31 | 8 | 10 | 8 | 10 | 20 |
| 6E008 | 21/04/2016 | environment | sawmill (soil) | S | NR | 21 | 22 | 18 | 27 | 9 | 15 | 9 | 13 | 8 |
| 1E026 | 17/09/2016 | environment | dwelling (dust) | R | TR46/Y121F/T289A | 26 | 20 | 12 | 32 | 8 | 11 | 12 | 9 | 20 |
| 1E027 | 27/09/2016 | environment | dwelling (dust) | R | TR34/L98H | 14 | 21 | 8 | 32 | 8 | 6 | 8 | 10 | 21 |
| 1E028 | 27/09/2016 | environment | dwelling (dust) | R | TR34/L98H | 14 | 21 | 8 | 32 | 8 | 6 | 8 | 10 | 21 |
| 1E001 | 04/10/2016 | clinic | cystic fibrosis | R | TR34/L98H | 14 | 21 | 8 | 31 | 8 | 6 | 8 | 10 | 20 |
| 1E029 | 18/10/2016 | environment | dwelling (dust) | R | F46Y/M172V/E427K | 10 | 16 | 10 | 18 | 11 | 11 | 7 | 5 | 5 |
| 1E007 | 14/11/2016 | clinic | cystic fibrosis | S | F46Y/M172V/N248T/D255E/E427K | 25 | 16 | 23 | 24 | 10 | 20 | 12 | 11 | 8 |
| 1E008 | 14/11/2016 | clinic | cystic fibrosis | R | F46Y/M172V/N248T/D255E/E427K | 25 | 16 | 23 | 24 | 10 | 20 | 12 | 11 | 8 |
| 1E023 | 28/11/2016 | environment | hospital (air) | R | TR34/L98H | 14 | 21 | 8 | 33 | 8 | 6 | 8 | 10 | 20 |
| 1E003 | 13/12/2016 | clinic | cystic fibrosis | R | TR34/L98H | 14 | 21 | 8 | 30 | 8 | 6 | 8 | 10 | 20 |
| 1E009 | 20/12/2016 | clinic | cystic fibrosis | R | G434 | 18 | 21 | 18 | 22 | 10 | 27 | 10 | 8 | 8 |
| 1E014 | 10/01/2017 | clinic | cystic fibrosis | R | TR34/L98H | 14 | 21 | 8 | 30 | 8 | 6 | 8 | 10 | 20 |
| 1E015 | 10/01/2017 | clinic | cystic fibrosis | R | TR34/L98H | 14 | 21 | 8 | 30 | 8 | 6 | 8 | 10 | 20 |
| 1E016 | 12/01/2017 | clinic | cystic fibrosis | R | TR34/L98H | 14 | 21 | 8 | 30 | 8 | 6 | 8 | 10 | 20 |
| 1E004 | 17/01/2017 | clinic | cystic fibrosis | R | TR34/L98H | 14 | 21 | 8 | 30 | 8 | 6 | 8 | 10 | 20 |
| 1E017 | 17/01/2017 | clinic | cystic fibrosis | R | TR34/L98H | 14 | 21 | 8 | 33 | 8 | 6 | 8 | 10 | 20 |
| 8E051 | 26/01/2017 | environment | hospital (air) | S | NR | 18 | 12 | 21 | 28 | 9 | 20 | 8 | 9 | 10 |
| 8E053 | 26/01/2017 | environment | hospital (air) | S | NR | 25 | 20 | 13 | 39 | 8 | 10 | 8 | 10 | 8 |
| 8E054 | 26/01/2017 | environment | hospital (air) | S | NR | 26 | 20 | 12 | 34 | 8 | 7 | 8 | 10 | 22 |
| 8E055 | 26/01/2017 | environment | hospital (air) | S | NR | 18 | 12 | 13 | 14 | 9 | 14 | 8 | 11 | 5 |
| 8E056 | 26/01/2017 | environment | hospital (air) | S | NR | 19 | 12 | 9 | 16 | 0 | 9 | 8 | 9 | 10 |
| 8E058 | 26/01/2017 | environment | hospital (air) | S | NR | 20 | 12 | 9 | 11 | 7 | 10 | 8 | 9 | 10 |
| 8E059 | 26/01/2017 | environment | hospital (air) | S | NR | 13 | 20 | 10 | 10 | 9 | 9 | 8 | 9 | 20 |
| 8E060 | 26/01/2017 | environment | hospital (air) | S | NR | 25 | 20 | 13 | 39 | 8 | 10 | 8 | 10 | 8 |
| 8E061 | 26/01/2017 | environment | hospital (air) | S | NR | 24 | 21 | 8 | 32 | 8 | 6 | 8 | 10 | 20 |
| 8E062 | 26/01/2017 | environment | hospital (air) | S | NR | 19 | 12 | 11 | 15 | 8 | 18 | 17 | 13 | 5 |
| 8E063 | 26/01/2017 | environment | hospital (air) | S | NR | 20 | 12 | 9 | 10 | 7 | 10 | 8 | 9 | 10 |
| 8E064 | 26/01/2017 | environment | hospital (air) | S | NR | 14 | 20 | 14 | 36 | 8 | 10 | 8 | 10 | 21 |
| 8E065 | 26/01/2017 | environment | hospital (air) | S | NR | 23 | 20 | 14 | 37 | 10 | 7 | 10 | 9 | 8 |
| 8E066 | 26/01/2017 | environment | hospital (air) | S | NR | 11 | 12 | 11 | 19 | 10 | 20 | 15 | 8 | 20 |
| 8E068 | 26/01/2017 | environment | hospital (air) | S | NR | 19 | 12 | 16 | 26 | 9 | 17 | 9 | 8 | 7 |
| 8E069 | 26/01/2017 | environment | hospital (air) | S | NR | 13 | 10 | 9 | 10 | 11 | 9 | 8 | 9 | 19 |
| 8E070 | 26/01/2017 | environment | hospital (air) | S | NR | 18 | 12 | 17 | 25 | 9 | 24 | 8 | 9 | 7 |
| 8E071 | 26/01/2017 | environment | hospital (air) | S | NR | 14 | 21 | 8 | 37 | 8 | 10 | 8 | 10 | 8 |
| 8E072 | 26/01/2017 | environment | hospital (air) | S | NR | 19 | 12 | 11 | 26 | 8 | 23 | 17 | 8 | 5 |
| 8E073 | 26/01/2017 | environment | hospital (air) | S | NR | 13 | 20 | 10 | 10 | 9 | 9 | 8 | 9 | 20 |
| 8E074 | 26/01/2017 | environment | hospital (air) | S | NR | 19 | 12 | 11 | 15 | 8 | 18 | 17 | 13 | 5 |
| 8E079 | 26/01/2017 | environment | hospital (air) | S | NR | 13 | 10 | 9 | 10 | 11 | 9 | 8 | 9 | 19 |
| 8E087 | 26/01/2017 | environment | hospital (air) | S | NR | 15 | 20 | 8 | 17 | 18 | 10 | 8 | 9 | 20 |
| 8E088 | 26/01/2017 | environment | hospital (air) | S | NR | 18 | 12 | 8 | 28 | 9 | 20 | 9 | 9 | 8 |
| 1E006 | 07/03/2017 | environment | market garden (soil) | R | TR34/L98H | 23 | 10 | 9 | 10 | 9 | 6 | 8 | 10 | 20 |
| 1E012 | 07/03/2017 | environment | market garden (soil) | R | TR34/L98H | 14 | 21 | 14 | 31 | 11 | 35 | 8 | 9 | 5 |
| 1E018 | 07/03/2017 | clinic | cystic fibrosis | R | TR34/L98H | 14 | 21 | 8 | 33 | 8 | 10 | 8 | 10 | 20 |
| 1E031 | 07/03/2017 | environment | market garden (soil) | R | TR34/L98H | 23 | 21 | 16 | 104 | 11 | 7 | 17 | 9 | 10 |
| 1E032 | 07/03/2017 | environment | market garden (soil) | R | TR34/L98H | 23 | 21 | 16 | 111 | 11 | 7 | 17 | 9 | 10 |
| 1E033 | 07/03/2017 | environment | market garden (soil) | R | TR34/L98H | 23 | 22 | 16 | 33 | 8 | 10 | 8 | 6 | 7 |
| 1E034 | 07/03/2017 | environment | market garden (soil) | R | TR34/L98H | 14 | 21 | 14 | 31 | 11 | 35 | 8 | 9 | 5 |
| 1E035 | 07/03/2017 | environment | market garden (soil) | R | TR34/L98H | 25 | 21 | 12 | 90 | 8 | 10 | 8 | 9 | 21 |
| 1E036 | 07/03/2017 | environment | market garden (soil) | R | TR34/L98H | 23 | 22 | 16 | 33 | 8 | 10 | 8 | 6 | 7 |
| 1E037 | 07/03/2017 | environment | market garden (soil) | R | TR34/L98H | 23 | 10 | 9 | 10 | 9 | 6 | 8 | 10 | 20 |
| 1E038 | 07/03/2017 | environment | market garden (soil) | R | TR34/L98H | 14 | 21 | 14 | 31 | 11 | 35 | 8 | 9 | 5 |
| 1E040 | 07/03/2017 | environment | market garden (soil) | R | TR46/Y121F/T289A | 25 | 20 | 12 | 45 | 8 | 6 | 12 | 10 | 9 |
| 1E041 | 07/03/2017 | environment | market garden (soil) | R | TR46/Y121F/T289A | 10 | 20 | 8 | 43 | 8 | 10 | 12 | 9 | 9 |
| 1E042 | 07/03/2017 | environment | market garden (soil) | R | TR46/Y121F/T289A | 25 | 20 | 12 | 45 | 8 | 6 | 12 | 10 | 9 |
| 1E043 | 07/03/2017 | environment | market garden (soil) | R | TR46/Y121F/T289A | 25 | 20 | 12 | 45 | 8 | 6 | 12 | 10 | 9 |
| 1E044 | 07/03/2017 | environment | market garden (soil) | R | TR34/L98H | 14 | 21 | 14 | 31 | 11 | 35 | 8 | 9 | 5 |
| 1E045 | 07/03/2017 | environment | market garden (soil) | R | TR34/L98H | 14 | 20 | 8 | 32 | 8 | 6 | 8 | 10 | 20 |
| 1E046 | 07/03/2017 | environment | market garden (soil) | R | TR34/L98H | 23 | 22 | 16 | 33 | 8 | 10 | 8 | 6 | 7 |
| 1E047 | 07/03/2017 | environment | market garden (soil) | R | TR34/L98H | 23 | 22 | 16 | 33 | 8 | 10 | 8 | 6 | 7 |
| 1E048 | 07/03/2017 | environment | market garden (soil) | R | TR34/L98H | 10 | 23 | 12 | 10 | 8 | 6 | 12 | 10 | 9 |
| 1E049 | 07/03/2017 | environment | market garden (soil) | R | TR34/L98H | 23 | 22 | 16 | 33 | 8 | 10 | 8 | 6 | 7 |
| 1E050 | 07/03/2017 | environment | market garden (soil) | R | TR34/L98H | 14 | 21 | 14 | 31 | 11 | 35 | 8 | 9 | 5 |
| 1E051 | 07/03/2017 | environment | market garden (soil) | R | TR34/L98H | 23 | 22 | 16 | 33 | 8 | 10 | 8 | 6 | 7 |
| 1E052 | 07/03/2017 | environment | market garden (soil) | R | TR34/L98H | 23 | 10 | 9 | 10 | 9 | 6 | 8 | 10 | 20 |
| 1E053 | 07/03/2017 | environment | market garden (soil) | R | TR34/L98H | 14 | 21 | 14 | 31 | 11 | 35 | 8 | 9 | 5 |
| 1E054 | 07/03/2017 | environment | market garden (soil) | R | TR46/Y121F/T289A | 25 | 20 | 9 | 10 | 8 | 11 | 12 | 9 | 20 |
| 1E055 | 07/03/2017 | environment | market garden (soil) | R | TR46/Y121F/T289A | 25 | 20 | 9 | 10 | 8 | 11 | 14 | 9 | 20 |
| 1E056 | 07/03/2017 | environment | market garden (soil) | R | TR34/L98H | 18 | 21 | 16 | 29 | 10 | 28 | 10 | 9 | 10 |
| 1E057 | 07/03/2017 | environment | market garden (soil) | R | TR46/Y121F/T289A | 25 | 20 | 9 | 10 | 8 | 11 | 14 | 9 | 20 |
| 1E058 | 07/03/2017 | environment | market garden (soil) | R | TR34/L98H | 14 | 21 | 14 | 31 | 11 | 35 | 8 | 9 | 5 |
| 1E059 | 07/03/2017 | environment | market garden (soil) | R | TR46/Y121F/T289A | 10 | 23 | 12 | 48 | 8 | 6 | 12 | 10 | 9 |
| 1E060 | 07/03/2017 | environment | market garden (soil) | R | TR34/L98H | 22 | 16 | 14 | 36 | 8 | 21 | 10 | 9 | 10 |
| 1E061 | 07/03/2017 | environment | market garden (soil) | R | TR46/Y121F/T289A | 10 | 23 | 12 | 48 | 8 | 6 | 12 | 10 | 9 |
| 1E062 | 07/03/2017 | environment | market garden (soil) | R | TR34/L98H | 14 | 21 | 14 | 31 | 11 | 35 | 8 | 9 | 5 |
| 1E063 | 07/03/2017 | environment | market garden (soil) | R | TR34/L98H | 23 | 21 | 16 | 103 | 11 | 7 | 17 | 9 | 10 |
| 1E064 | 07/03/2017 | environment | market garden (soil) | R | TR34/L98H | 23 | 21 | 16 | 103 | 11 | 7 | 17 | 9 | 10 |
| 1E065 | 07/03/2017 | environment | market garden (soil) | R | TR34/L98H | 25 | 21 | 12 | 90 | 8 | 10 | 8 | 9 | 21 |
| 1E066 | 07/03/2017 | environment | market garden (soil) | R | TR34/L98H | 23 | 21 | 16 | 101 | 11 | 7 | 17 | 9 | 10 |
| 1E067 | 07/03/2017 | environment | market garden (soil) | R | TR34/L98H | 23 | 21 | 16 | 104 | 11 | 7 | 17 | 17 | 10 |
| 1E073 | 07/03/2017 | environment | market garden (soil) | R | TR34/L98H | 14 | 21 | 8 | 28 | 12 | 21 | 10 | 9 | 5 |
| 4E007 | 07/03/2017 | environment | market garden (soil) | S | NR | 10 | 16 | 10 | 26 | 11 | 8 | 7 | 5 | 6 |
| 4E008 | 07/03/2017 | environment | market garden (soil) | S | NR | 14 | 20 | 11 | 35 | 8 | 10 | 8 | 10 | 20 |
| 4E009 | 07/03/2017 | environment | market garden (soil) | S | NR | 10 | 16 | 10 | 25 | 11 | 8 | 7 | 5 | 6 |
| 4E010 | 07/03/2017 | environment | market garden (soil) | S | NR | 10 | 16 | 10 | 18 | 10 | 13 | 7 | 5 | 6 |
| 4E011 | 07/03/2017 | environment | market garden (soil) | S | NR | 13 | 24 | 10 | 47 | 9 | 14 | 8 | 7 | 21 |
| 4E012 | 07/03/2017 | environment | market garden (soil) | S | NR | 10 | 16 | 10 | 28 | 11 | 8 | 7 | 5 | 6 |
| 4E013 | 07/03/2017 | environment | market garden (soil) | S | NR | 13 | 10 | 9 | 10 | 10 | 9 | 8 | 9 | 19 |
| 4E014 | 07/03/2017 | environment | market garden (soil) | S | NR | 21 | 12 | 10 | 36 | 39 | 31 | 8 | 9 | 7 |
| 4E015 | 07/03/2017 | environment | market garden (soil) | S | NR | 10 | 15 | 10 | 19 | 12 | 12 | 7 | 5 | 6 |
| 4E016 | 07/03/2017 | environment | market garden (soil) | S | NR | 18 | 9 | 8 | 22 | 8 | 21 | 8 | 9 | 7 |
| 4E017 | 07/03/2017 | environment | market garden (soil) | S | NR | 18 | 12 | 11 | 13 | 8 | 7 | 8 | 9 | 5 |
| 4E018 | 07/03/2017 | environment | market garden (soil) | S | NR | 23 | 10 | 9 | 29 | 9 | 7 | 12 | 9 | 12 |
| 4E020 | 07/03/2017 | environment | market garden (soil) | S | NR | 13 | 10 | 9 | 24 | 10 | 9 | 8 | 11 | 19 |
| 4E021 | 07/03/2017 | environment | market garden (soil) | S | NR | 24 | 20 | 8 | 33 | 7 | 6 | 8 | 10 | 20 |
| 1E068 | 10/03/2017 | environment | market garden (soil) | R | TR34/L98H | 14 | 20 | 8 | 40 | 8 | 7 | 8 | 10 | 14 |
| 1E069 | 10/03/2017 | environment | market garden (soil) | R | TR34/L98H | 14 | 21 | 8 | 31 | 8 | 6 | 8 | 10 | 20 |
| 1E070 | 10/03/2017 | environment | market garden (soil) | R | TR34/L98H | 14 | 21 | 8 | 31 | 8 | 6 | 8 | 10 | 20 |
| 1E085 | 10/03/2017 | environment | market garden (soil) | R | wild type | 14 | 20 | 8 | 59 | 8 | 9 | 8 | 10 | 20 |
| 4E022 | 10/03/2017 | environment | market garden (soil) | S | NR | 23 | 20 | 29 | 36 | 10 | 7 | 66 | 25 | 5 |
| 4E023 | 10/03/2017 | environment | market garden (soil) | S | NR | 13 | 22 | 9 | 40 | 8 | 10 | 8 | 9 | 40 |
| 4E024 | 10/03/2017 | environment | market garden (soil) | S | NR | 14 | 10 | 12 | 34 | 8 | 9 | 8 | 12 | 10 |
| 4E025 | 10/03/2017 | environment | market garden (soil) | S | NR | 18 | 18 | 15 | 34 | 10 | 27 | 17 | 9 | 8 |
| 4E026 | 10/03/2017 | environment | market garden (soil) | S | NR | 10 | 16 | 10 | 19 | 11 | 11 | 7 | 5 | 5 |
| 4E027 | 10/03/2017 | environment | market garden (soil) | S | NR | 25 | 20 | 8 | 33 | 8 | 10 | 8 | 10 | 31 |
| 1E010 | 28/04/2017 | clinic | cystic fibrosis | R | wild type | 18 | 12 | 13 | 21 | 9 | 12 | 8 | 9 | 5 |
| 1E011 | 16/05/2017 | clinic | cystic fibrosis | R | wild type | 18 | 12 | 13 | 25 | 9 | 12 | 8 | 9 | 5 |
| 1E019 | 16/05/2017 | clinic | cystic fibrosis | R | TR34/L98H | 23 | 21 | 16 | 17 | 8 | 7 | 5 | 9 | 10 |
| 1E025 | 29/05/2017 | environment | hospital (air) | R | TR34/L98H | 23 | 21 | 16 | 26 | 8 | 7 | 5 | 9 | 10 |
| 4E005 | 20/06/2017 | clinic | cystic fibrosis | S | NR | 25 | 20 | 8 | 10 | 9 | 21 | 9 | 10 | 5 |
| 1E013 | 11/07/2017 | clinic | cystic fibrosis | R | TR34/L98H | 14 | 21 | 8 | 30 | 8 | 6 | 8 | 10 | 20 |
| 1E005 | 12/07/2017 | clinic | cystic fibrosis | R | TR34/L98H | 14 | 21 | 8 | 30 | 8 | 6 | 8 | 10 | 20 |
| 1E071 | 14/09/2017 | clinic | cystic fibrosis | R | TR34/L98H | 14 | 21 | 8 | 30 | 8 | 6 | 8 | 10 | 20 |
| 4E006 | 28/09/2017 | clinic | cystic fibrosis | S | NR | 19 | 19 | 18 | 42 | 10 | 32 | 26 | 11 | 5 |
| 1E072 | 04/10/2017 | clinic | cystic fibrosis | R | TR34/L98H | 23 | 21 | 16 | 103 | 8 | 7 | 5 | 9 | 10 |
| 1E074 | 17/10/2017 | environment | hospital (air) | R | TR34/L98H | 14 | 21 | 8 | 31 | 8 | 6 | 8 | 10 | 20 |
| 1E081 | 29/10/2017 | clinic | icu | R | wild type | 13 | 20 | 11 | 26 | 8 | 7 | 8 | 10 | 20 |
| 1E075 | 10/11/2017 | clinic | cystic fibrosis | R | TR34/L98H | 14 | 21 | 8 | 30 | 8 | 6 | 8 | 10 | 20 |
| 1E076 | 17/11/2017 | environment | hospital (air) | R | TR34/L98H | 14 | 21 | 8 | 33 | 8 | 10 | 8 | 10 | 20 |
| 1E077 | 17/11/2017 | environment | hospital (air) | R | TR34/L98H | 14 | 21 | 8 | 33 | 8 | 10 | 8 | 10 | 20 |
| 1E078 | 17/11/2017 | environment | hospital (air) | R | TR34/L98H | 14 | 21 | 8 | 30 | 8 | 6 | 8 | 10 | 20 |
| 1E079 | 21/11/2017 | environment | hospital (air) | R | TR34/L98H | 14 | 21 | 8 | 31 | 8 | 6 | 8 | 10 | 20 |
| 1E080 | 21/11/2017 | environment | hospital (air) | R | TR34/L98H | 14 | 21 | 8 | 32 | 8 | 6 | 8 | 10 | 21 |
| 1E082 | 05/12/2017 | clinic | cystic fibrosis | R | G434 | 18 | 21 | 18 | 22 | 10 | 27 | 10 | 8 | 8 |
| 1E086 | 27/03/2018 | clinic | cystic fibrosis | R | TR34/L98H | 23 | 21 | 16 | 122 | 8 | 7 | 5 | 9 | 10 |
| 1E087 | 10/04/2018 | environment | hospital (air) | R | TR34/L98H | 14 | 21 | 8 | 30 | 8 | 6 | 8 | 10 | 20 |
| 1E088 | 27/06/2018 | environment | hospital (air) | R | TR34/L98H | 25 | 21 | 8 | 32 | 8 | 7 | 8 | 10 | 19 |
| 2E036 | 27/06/2018 | environment | hospital (air) | S | NR | 18 | 12 | 12 | 23 | 21 | 19 | 14 | 8 | 7 |
| 2E037 | 27/06/2018 | environment | hospital (air) | S | NR | 18 | 19 | 8 | 26 | 9 | 20 | 9 | 9 | 5 |
| 2E038 | 27/06/2018 | environment | hospital (air) | S | NR | 23 | 23 | 15 | 39 | 10 | 43 | 9 | 9 | 8 |
| 2E039 | 27/06/2018 | environment | hospital (air) | S | NR | 14 | 20 | 12 | 43 | 8 | 7 | 8 | 12 | 33 |
| 2E040 | 27/06/2018 | environment | hospital (air) | S | NR | 18 | 12 | 7 | 28 | 9 | 19 | 8 | 8 | 7 |
| 2E042 | 27/06/2018 | environment | hospital (air) | S | NR | 25 | 20 | 8 | 10 | 9 | 21 | 9 | 10 | 5 |
| 2E041 | 02/07/2018 | environment | hospital (air) | S | NR | 15 | 20 | 8 | 26 | 8 | 7 | 8 | 10 | 20 |
| XE114 | 02/07/2018 | environment | hospital (air) | S | NR | 13 | 20 | 11 | 32 | 7 | 10 | 8 | 10 | 20 |
| XE115 | 02/07/2018 | environment | hospital (air) | S | NR | 23 | 19 | 17 | 27 | 8 | 15 | 5 | 16 | 11 |
| 2E019 | 06/09/2018 | environment | hospital (air) | R | TR34/L98H | 25 | 11 | 12 | 85 | 8 | 8 | 8 | 10 | 8 |
| 2E022 | 13/09/2018 | clinic | cystic fibrosis | R | F46Y/M172V/N248T/D255E/E427K | 25 | 16 | 23 | 24 | 10 | 20 | 12 | 11 | 8 |
| 2E027 | 27/09/2018 | clinic | cystic fibrosis | R | TR34/L98H | 24 | 20 | 18 | 35 | 8 | 7 | 8 | 14 | 5 |
| 2E025 | 05/10/2018 | clinic | hematology | R | TR34/L98H | 23 | 21 | 16 | 24 | 8 | 7 | 5 | 9 | 10 |
| 2E026 | 12/10/2018 | clinic | cystic fibrosis | R | F46Y/M172V/N248T/D255E/E427K | 25 | 16 | 22 | 24 | 10 | 20 | 12 | 11 | 8 |
| 2E029 | 23/10/2018 | environment | dwelling (dust) | R | TR34/L98H | 25 | 20 | 19 | 35 | 8 | 31 | 8 | 14 | 5 |
| 2E086 | 18/12/2018 | environment | hospital (air) | S | NR | 25 | 22 | 18 | 27 | 8 | 26 | 10 | 9 | 5 |
| 2E088 | 18/12/2018 | environment | hospital (air) | S | NR | 25 | 22 | 19 | 27 | 8 | 26 | 10 | 9 | 5 |
| 2E090 | 18/12/2018 | environment | hospital (air) | S | NR | 25 | 20 | 11 | 34 | 8 | 9 | 2 | 10 | 26 |
| 2E093 | 18/12/2018 | environment | hospital (air) | S | NR | 19 | 19 | 8 | 15 | 8 | 21 | 8 | 9 | 5 |
| 2E096 | 18/12/2018 | environment | hospital (air) | S | NR | 15 | 23 | 8 | 10 | 12 | 19 | 13 | 9 | 5 |
| 2E097 | 18/12/2018 | environment | hospital (air) | S | NR | 18 | 12 | 13 | 29 | 9 | 21 | 8 | 9 | 5 |
| 2E098 | 18/12/2018 | environment | hospital (air) | S | NR | 18 | 12 | 13 | 14 | 21 | 12 | 8 | 9 | 5 |
| 5E003 | 18/12/2018 | environment | hospital (air) | S | NR | 23 | 19 | 16 | 27 | 9 | 7 | 9 | 9 | 5 |
| 5E004 | 18/12/2018 | environment | hospital (air) | S | NR | 21 | 25 | 19 | 27 | 11 | 7 | 21 | 10 | 8 |
| 5E005 | 18/12/2018 | environment | hospital (air) | S | NR | 17 | 12 | 12 | 14 | 9 | 19 | 8 | 8 | 5 |
| 5E006 | 18/12/2018 | environment | hospital (air) | S | NR | 18 | 12 | 8 | 28 | 9 | 20 | 9 | 9 | 5 |
| 5E009 | 18/12/2018 | environment | hospital (air) | S | NR | 14 | 21 | 11 | 27 | 8 | 7 | 8 | 10 | 31 |

NR : Not realized
